# Telbivudine for the treatment of chronic hepatitis B in HBeAg-positive patients in China: a health economic analysis

**DOI:** 10.1186/s40064-016-3404-x

**Published:** 2016-10-05

**Authors:** S. Banerjee, P. Gunda, R. F. Drake, K. Hamed

**Affiliations:** 1 Novartis Healthcare Pvt. Ltd., Hyderabad, India; 2 Novartis Pharma AG, Basel, Switzerland; 3 Novartis Pharmaceuticals Corporation, East Hanover, NJ USA

**Keywords:** Cost effectiveness, Chronic hepatitis B, Renal impairment, Telbivudine

## Abstract

**Background:**

Nucleos(t)ide analogs (NUCs) are the standard of care for chronic hepatitis B (CHB). The present analysis aimed to determine the cost effectiveness of NUCs in Chinese healthcare settings.

**Methods:**

A Markov model was used to simulate two therapeutic strategies for a hypothetical patient cohort diagnosed with hepatitis B e antigen-positive CHB, unwilling or unable to receive interferon therapy, and about to start treatment with any NUC. The first strategy included NUC monotherapy without sequencing (telbivudine [LDT], entecavir [ETV], tenofovir [TDF], lamivudine [LAM], adefovir dipivoxil [ADV], and combination therapies of either LDT and ADV or LDT and TDF, followed by best supportive care [BSC]). The second strategy included sequential therapies of individual NUCs: LAM → ADV, ADV → LAM, LDT → ADV, and ETV → ADV, followed by BSC. The analysis included two scenarios: with and without costs due to nephrotoxicity. Renal impact was quantified as costs alone, without consideration for quality of life decrements.

**Results:**

When renal impact was not considered, without treatment sequencing, LDT was cost effective compared with other NUCs. Amongst the strategies with sequencing, LDT → ADV was cost effective. The results were similar when renal impact was considered. However, LDT strategy demonstrated better cost effectiveness. In probabilistic sensitivity analysis, in both scenarios, LDT → ADV sequence was cost effective with 51 % probability even at willingness to pay of $20,000.

**Conclusion:**

Use of LDT, as compared with other NUCs, is cost effective in CHB treatment in Chinese healthcare settings. Considering the detrimental renal impact, overall costs for all treatment options were increased. However, the increase for LDT was comparatively small.

## Background

Approximately 240 million people worldwide are chronically infected with the hepatitis B virus (HBV) (World Health Organization 2015). China has the highest burden, with an estimated 100 million people with chronic hepatitis B (CHB), translating into approximately 300,000 annual deaths from HBV-related liver complications (Vellozzi and Averhoff 2016) such as end-stage liver disease and hepatocellular carcinoma (HCC) (Ng et al. 2013; Robotin 2011). Eradication of HBV is a national priority in China (Yu et al. 2014), and various healthcare programs have been designed to address this imperative. In 2010, the Chinese Ministry of Science and Technology commissioned a project to evaluate the economic burden of HBV-related diseases (National Health and Family Planning Commission of the People’s Republic of China 2015).

Nucleos(t)ide analogs (NUCs) represent the mainstay of pharmacological treatment for CHB (Wong et al. 2014). Five NUCs, namely telbivudine [LDT], entecavir [ETV], tenofovir [TDF], lamivudine [LAM], and adefovir dipivoxil [ADV], are currently being used for the treatment of CHB worldwide (Deray et al. 2015). NUCs primarily act by suppressing HBV replication, thereby minimizing the risk of liver disease progression and subsequent complications, including hepatic decompensation and HCC, in both pre-cirrhotic and cirrhotic patients (Fung et al. 2011). Considering that NUC treatment does not eradicate the virus, most patients require long-term treatment. NUCs are generally safe and well tolerated, but nephrotoxic effects have been reported with long-term treatment. Renal toxicity develops because of accumulation of NUC metabolites in renal tubular cells. Clinical evidence suggests that nephrotoxicity is more frequent with ADV, followed by TDF (Deray et al. 2015). Nephrotoxicity clinically manifests as a decrease in glomerular filtration rate (GFR) and is more common in patients older than 50 years and those with baseline renal insufficiency, hypertension, and/or diabetes mellitus. The Chinese guidelines provide recommendations on the selection of effective treatments for CHB patients, but these are not driven by pharmacoeconomic evidence (Chinese Society of Hepatology and Chinese Society of Infectious Diseases and Chinese Medical Association 2011; Zhang et al. 2015). In spite of extensive use in clinical practice, the cost of NUCs is the primary factor that drives their real-world use as well as adherence among patients (Fung et al. 2011). This is particularly seen in countries with limited healthcare resources, such as China. The Chinese healthcare setting is evolving from a resource-constrained scenario to a modern healthcare framework (Blumenthal and Hsiao 2015). In such a setting, treatment strategies driven by cost-effectiveness evidence may help optimize case management of CHB in hepatitis B e antigen (HBeAg)-positive patients in China.

Multiple economic analyses have been conducted to compare the available NUCs in various settings (Almeida et al. 2012; He et al. 2012; Spackman and Veenstra 2008; Wu et al. 2010; Zhang et al. 2015). Nevertheless, the previous studies did not model the effects of renal impairment, resistance, sequencing of treatment, or long-term disease progression. Hence, cost-effectiveness analyses are needed to determine the most cost-effective NUC(s) for CHB treatment. The objective of the current analysis was to evaluate the cost effectiveness of NUCs using two treatment strategies from the perspective of the Chinese healthcare system.

## Methods

### Treatment strategies

The first strategy included NUC treatment options without sequencing: LDT, ETV, TDF, LAM, ADV, combination therapies of LDT and ADV or LDT and TDF, each followed by best supportive care (BSC). The second strategy included treatment sequencing: LAM → ADV, ADV → LAM, LDT → ADV, and ETV → ADV, followed by BSC. It was assumed that patients with HBV resistance to the first NUC would be switched to the second NUC, and subsequently to BSC, in the second strategy.

### Model structure and description

**Fig. 1:**
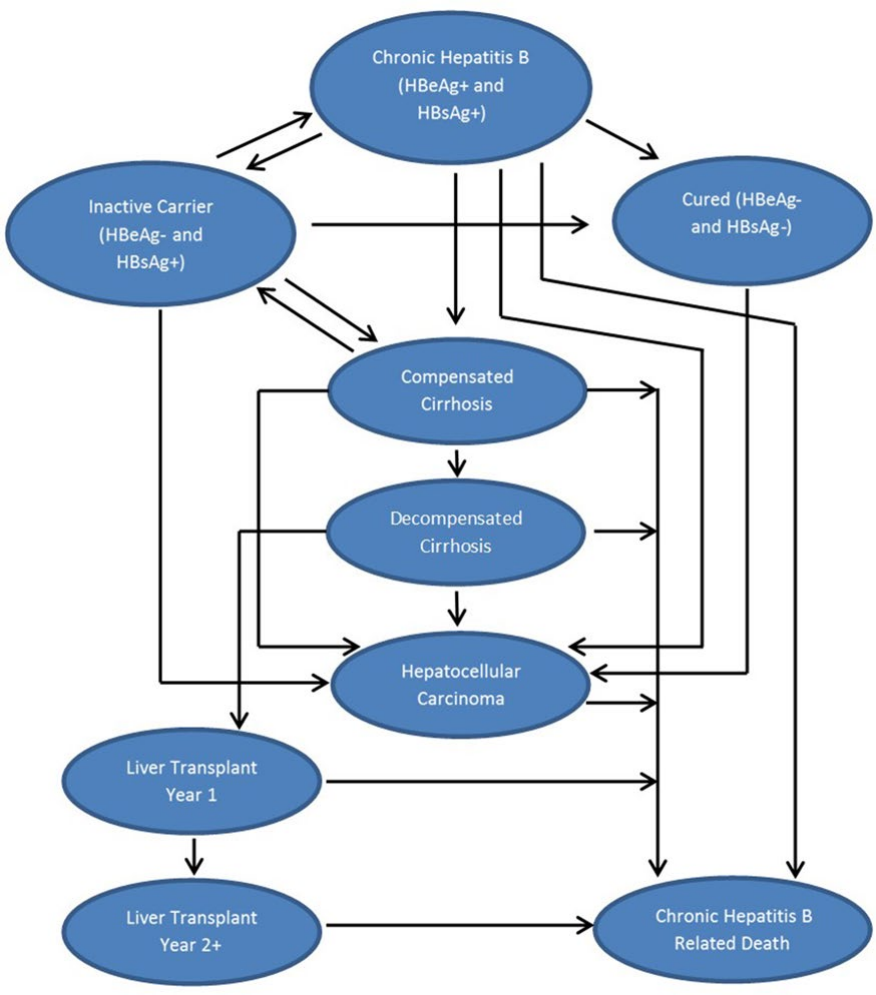
Schematic representation of the Markov model structure

A de novo Markov transition model was developed in MS Excel 2010^®^ to estimate the cost effectiveness of NUCs in the treatment of CHB. This model assumed that patients were always in one of the finite number of health states, referred to as Markov states. Patients were transitioned among the Markov states according to a set of transition probabilities that depended only on the current health state. Patients stayed in the same health state but moved to the next-line treatment if they developed resistance to a treatment. The present health economic model consisted of the following eight health states (Fig. 1):

1. Cured (generally assumed as HBeAg negative or hepatitis B surface antigen [HBsAg] negative and not corresponding to the clinical definition of cure)
2. Inactive carrier (HBsAg positive and HBeAg negative)
3. CHB
4. Compensated cirrhosis (CC)
5. Decompensated cirrhosis (DC)
6. HCC
7. Liver transplant (LT) year 1 (LT Year 1; in the year of transplantation)
8. Liver transplant year 2 onwards (LT Year 2+; after first year of transplantation)

Two additional absorbing health states were considered: CHB-related death and death due to non-CHB causes. Transitions among the health states were determined based on disease progression and treatment efficacy (Tables 1, 2). The cost-effectiveness analysis used a cycle length of 1 year and followed the patients for their entire life. The underlying assumption while constructing the Markov trace for CHB patients was that HBV resistance would not develop in patients who move to the inactive carrier state (with typically low HBV DNA levels) in the same cycle and/or who remain in the inactive carrier state. Substantial clinical evidence indicates that on-treatment HBV DNA levels are predictive of virologic resistance in CHB patients (Biazar et al. 2015; Chang 2009). Low rates of resistance were reported during the first and second years of telbivudine therapy in patients who achieved undetectable serum HBV DNA levels at treatment week 24 (Liaw 2009; Liu 2013). Furthermore, undetectable HBV DNA after 2 years of telbivudine treatment was also reported to be associated with low telbivudine resistance (Zeuzem et al. 2009).

**Table 1:** Treatment-specific transition probabilities for CHB to cured and CHB to inactive carrier transitions

Treatment strategy	CHB to cured and CHB to inactive carrier transitions				First-year reactivation probability (transition from inactive carrier to CHB)	
	CHB to cured (%)	Source	CHB to inactive carrier (%)	Source		Source
LAM	0.7	Zhang et al. (2015)	24.1	Zhang et al. (2015)	0.175	Hou et al. (2008, 2015)
LDT	0.7		33.3		0.075	
ADV	1.3		18.3		0.04	Hou et al. (2015)
ETV	1.4		25.6		0	Yuen et al. (2011)
TDF	0.7	Conservative assumption made for the least effective treatment amongst monotherapies (i.e. LAM/LDT)	15.5	Hou et al. (2008)	0	Hou et al. (2015)
LDT + ADV	0.7	Sun et al. (2014)	35.7	Sun et al. (2014) and Zhang et al. (2015) [derived using LDT and LDT + ADV ratio from Sun et al. (2014)]	0.01	Sun et al. (2014) and Zhang et al. (2015) [derived using LDT and LDT + ADV ratio from Sun et al. (2014)]
LDT + TDF	4.4	Piratvisuth et al. (2013) and Zhang et al. (2015) [derived using LDT and LDT + TDF ratio from Piratvisuth et al. (2013)]	6.1	Piratvisuth et al. (2013) and Zhang et al. (2015) [derived using LDT and LDT + TDF ratio from Piratvisuth et al. (2013)]	0	Piratvisuth et al. (2013)
No treatment	0.7	Assumed to be equal to the least effective treatment (i.e. LDT/LAM)	9.0	Shepherd et al. (2006)	0.03	Shepherd et al. (2006)

*ADV* adefovir dipivoxil, *BSC* best supportive care, *CHB* chronic hepatitis B, *ETV* entecavir, *LAM* lamivudine, *LDT* telbivudine, *TDF* tenofovir

**Table 2:** Other transition probabilities used in model

Treatment independent transitions		
Transition	Probability	Source
Inactive carrier to cured	0.020	Shepherd et al. (2006)
Inactive carrier to CHB	0.030	
Inactive carrier to CC	0.009	
Cured to HCC	0.00005	Zhang et al. (2015)
Inactive carrier to HCC	0.002	
CHB to CC	0.010	
CHB to HCC	0.004	
CHB to Dead	0.009	
CC to inactive carrier	0.090	Shepherd et al. (2006)
CC to HCC	0.018	Zhang et al. (2015)
CC to dead	0.025	
DC to HCC	0.091	
DC to LT Year 1	0.050	
HCC to Dead	0.520	

Treatment specific other transitions		
Transition	Probability for NUCs	Source
CC to DC	0.00936 (RR = 0.36)	Shepherd et al. (2006)
DC to dead	0.052 (RR = 0.50)	
LT Year 1 to dead	0.012 (RR = 0.1)	
LT Year 2+ to dead	0.0057 (RR = 0.1)	

Transition	Probability for BSC	Source
CC to DC	0.026	Zhang et al. (2015)
DC to dead	0.104	
LT Year 1 to dead	0.120	
LT Year 2+ to dead	0.057	Shepherd et al. (2006)

*ADV* adefovir dipivoxil, *BSC* best supportive care, *CC* compensated cirrohoiss, *CHB* chronic hepatitis B, *DC* decompensated cirrhosis, *ETV* entecavir, *HCC* hepatocellular carcinoma, *LAM* lamivudine, *LDT* telbivudine, *LT* liver transplant, *NUCs* nucleos(t)ide analogs, *RR* relative risk, *TDF* tenofovir

In the deterministic analysis, total discounted costs and total discounted quality-adjusted life-years (QALYs) were estimated. Since multiple treatment strategies were compared, results were depicted on a cost-effectiveness frontier. Apart from the deterministic analysis, a probabilistic sensitivity analysis (PSA) was also performed. For the PSA, β distribution was used for transition probabilities, treatment effects, reactivation rates, utilities, and viral resistance, whereas γ distribution was used for average eGFR and costs, and normal distribution for change in eGFR.

Patients discontinued the treatment if 1 year had elapsed after converting to HBeAg-negative status as per clinical guidelines (Sarin et al. 2016). The analyses involved two scenarios; the first scenario did not include the detrimental renal impact of the NUCs, while the second scenario included the nephrotoxic effects of the treatments. Renal impact was quantified in terms of costs only, and any quality of life decrements due to renal impact were not considered in the analysis.

### Model settings

#### Population

The model simulated the experiences of a hypothetical cohort of patients who were diagnosed with HBeAg-positive CHB, were unwilling or unable to receive interferon therapy, and were about to start treatment with one of the NUCs. If patients developed resistance to a treatment, they were assumed to stay in the same health state but move to the next-line treatment. The starting age of the cohort was 31 years, and 75 % were males. The model was developed with a cycle length of 1 year and followed patients for a lifetime.

#### Discounting

Costs and outcomes were discounted at 3.5 % per annum.

#### Length of consolidation treatment after HBeAg seroconversion

The model determined the impact on cost-effectiveness results when the treatment was continued for a minimum of 1 year after HBeAg seroconversion (as per the Asian-Pacific clinical practice guidelines on the management of hepatitis B (Sarin et al. 2016)). Although HBsAg seroclearance is the ideal endpoint, it is only achievable in up to 12 % of patients after long-term NUC treatment (Yuen et al. 2016). Therefore, finite therapy (treatment of 1 year) based on HBeAg seroconversion, which represents the current standard-of-care in China, was employed in the model.

#### Perspective

The model adapted the perspectives of the Chinese healthcare settings.

#### Model inputs

Efficacy values specific to the Chinese population were included wherever possible for all the model inputs.

#### Treatment regimens

Various treatment strategies currently employed for the management of CHB patients in China were used (Zhang et al. 2015) (Table 3).

**Table 3:** Treatment regimens used in the model

Treatment strategy	Treatment explanation (“A → B” indicates after developing resistance to treatment A, patients move to treatment B; “+” indicates combination therapy)
BSC	No antiviral drug treatment
LAM → BSC	LAM as first-line therapy, followed by BSC as second- and third-line therapy
LDT → BSC	LDT as first-line therapy, followed by BSC as second- and third-line therapy
ADV → BSC	ADV as first-line therapy, followed by BSC as second- and third-line therapy
ETV → BSC	ETV as first-line therapy, followed by BSC as second- and third-line therapy
TDF → BSC	TDF as first-line therapy, followed by BSC as second- and third-line therapy
LDT + ADV → BSC	Combination therapy of LDT and ADV as first-line therapy, followed by BSC as second- and third-line therapy
LDT + TDF → BSC	Combination therapy of LDT and TDF as first-line therapy, followed by BSC as second- and third-line therapy
LAM → ADV → BSC	LAM as first-line therapy, followed by ADV as second-line therapy and BSC as third-line therapy
ADV → LAM → BSC	ADV as first-line therapy, followed by LAM as second-line therapy and BSC as third-line therapy
LDT → ADV → BSC	LDT as first-line therapy, followed by ADV as second-line therapy and BSC as third-line therapy
ETV → ADV → BSC	ETV as first-line therapy, followed by ADV as second-line therapy and BSC as third-line therapy

*ADV* adefovir dipivoxil, *BSC* best supportive care, *ETV* entecavir, *LAM* lamivudine, *LDT* telbivudine, *TDF* tenofovir

#### Efficacy inputs

HBsAg and HBeAg seroconversions with all treatments were obtained from the published literature (Table 3). The model included reactivation rate, i.e. patients transitioning from the inactive carrier state (HBeAg negative and HBsAg positive) to CHB status (HBeAg positive and HBsAg positive). This reactivation rate was modeled only for year 1, during which it is expected to be maximum. These values were obtained from the published literature (Table 3). It was assumed that the first NUC treatment would lower the occurrence of cirrhosis by 40 % in comparison with BSC (Shepherd et al. 2006). The resistance rates for the NUCs used in the model, along with their sources, are provided in Table 4 (Liaw et al. 2009; Zhang et al. 2015).

**Table 4:** Resistance profiles of antiviral therapies

Resistance profiles							
Year	ADV	LDT	LAM	ETV	TDF	LDT + ADV	LDT + TDF
1	0 %	3 %	9 %	0 %	0 %	3 %	3 %
2	2 %	15 %	22 %	1 %	0 %	15 %	15 %
3	5 %	15 %	22 %	3 %	0 %	15 %	15 %
4	8 %	15 %	22 %	3 %	0 %	15 %	15 %
5	8 %	15 %	22 %	3 %	0 %	15 %	15 %
	GLOBE trial (Liaw et al. 2009)			Zhang et al. (2015)	Piratvisuth et al. (2013)	Conservative assumption of resistance of same as LDT	Conservative assumption of resistance of same as LDT

*ADV* adefovir dipivoxil, *ETV* entecavir, *LAM* lamivudine, *LDT* telbivudine, *TDF* tenofovir

#### Long-term disease progression

For BSC, long-term transition to more severe health states (as stated below) were obtained from a recent publication (Zhang et al. 2015). As long-term data on the effects of NUCs were not available, the transitions below were considered to be similar to those with BSC.

- CC to DC
- DC to dead
- LT Year 1 to dead
- LT Year 2+ to dead

#### Renal impairment inputs

The model also captured the effects of long-term use of NUCs on renal function. The annual changes in eGFR by treatment are listed in Table 5, along with their sources. In the analysis, an average of available data was extrapolated for follow-up years. Annual changes in eGFR were used to estimate the eGFR of a cohort at the end of each cycle for a particular treatment regimen, and this eGFR was then used to estimate renal costs. All patients were assumed to start with a chronic kidney disease (CKD) stage 1 and an eGFR of 108.1 mL/min/1.73 m^2^ (i.e. normal eGFR) (Tsai et al. 2016). When eGFR is less than or equal to 15 mL/min/1.73 m^2^, the patient would be considered to go on dialysis and remain on dialysis until renal transplantation (Tattersall et al. 2011). In the base case, the waiting time for a kidney transplant was assumed to be 3 years, and post-transplantation the patient was assumed to move to CKD stage 1.

**Table 5:** Changes in eGFR by year for various treatment options (variation per year compared with previous year)

Treatment	Year 1	Year 2	Year 3	Year 4	Year 5	Source	Year >5
TDF	−6.40	0.70	0.70	0.70	0.70	Tsai et al. (2016)	−0.72
LDT	9.57	5.86	10.81	10.81	10.81	Qi et al. (2015)	9.57
ETV	0.00	1.99	−3.27	−3.27	−3.27	Qi et al. (2015)	−1.57
LAM	−4.72	−5.40	−2.29	−2.29	−2.29	Qi et al. (2015)	−3.40
ADV	−6.92	−4.72	−3.74	−3.74	−3.74	Qi et al. (2015)	−4.57
LDT + ADV	9.57	5.86	10.81	10.81	10.81	Assumed to be same as LDT	9.57
LDT + TDF	9.57	5.86	10.81	10.81	10.81		9.57
BSC	−0.69	−0.38	−0.73	−0.73	−0.73	Qi et al. (2015)	−0.65

eGFR was measured in mL/min/1.73 m^2^. For the eGFR changes, for each treatment, the last available observations were carried forward till year 5

*ADV* adefovir dipivoxil, *BSC* best supportive care, *eGFR* estimated glomerular filtration rate, *ETV* entecavir, *LAM* lamivudine, *LDT* telbivudine, *TDF* tenofovir

#### Utility inputs

Utilities were assigned for each health state (Table 6). Utility inputs for the health states were derived from a previous study by Levy et al. (2008) that evaluated utilities using a standard gamble technique in the Chinese population.

**Table 6:** Cost inputs used in the China seroconversion model

Cost parameter	Annual cost ($)	Source	Utility	Source
*Heath state costs*				
Cured (HBsAg negative)	1315.9	Zhang et al. (2015)	0.710	Levy et al. (2008)
Inactive carrier	2237.5		0.710	
Chronic hepatitis B	2237.5		0.520	
Compensated cirrhosis	3468.5		0.570	
Decompensated cirrhosis	6449.3		0.260	
Hepatocellular carcinoma	9179.5		0.310	
Liver transplant year 1	57,765.5		0.410	
Liver transplant year 2+	9626.9		0.550	
*Drug costs*				
LAM	710.41	IMS PADDS database, cost of LAM is derived from Zhang et al. (2015); inflation adjusted		
ADV	579.54			
LDT	1132.41			
ETV	1073.10			
TDF	2636.08			
LDT + ADV	1711.95			
LDT + TDF	3768.49			
BSC	NA			
Renal Drug Cost (tacrolimus 0.25 mg daily; MMF 2 g daily, and prednisolone 30 mg daily for 90 days)	2103	IMS PADDS database 2015		
*Procedure costs*				
Dialysis (for CKD 5 patients)	17,580	Dialysis cost of $17,280 (Liu 2013) (adjusted for inflation) + monitoring cost of $300		
Transplant (for CKD 5 patients)	11,825	Transplant cost of $11,525 (Zhao et al. 2012) (adjusted for inflation) + monitoring cost of $300		
Cost per hospital visit (assumed as unit cost of physician visit in China)*	25	Chinese medical news website (Woodhead 2015)		
*Annual examination costs*				
For all antiviral therapies	169.01	Zhang et al. (2015)		
For BSC	175			
Cost for evaluation of new patient	169			

*ADV* adefovir dipivoxil, *BSC* best supportive care, *CKD* chronic kidney disease, *ETV* entecavir, *HBsAg* hepatitis B surface antigen, *LAM* lamivudine, *LDT* telbivudine, *MMF* mycophenolate mofetil, *NA* not applicable, *TDF* tenofovir

* This cost was used to calculate disease monitoring costs. For CKD stages 1 and 2, 3, and 4 and 5, we assumed 4, 8, and 12 yearly visits, respectively

#### Mortality inputs

A life table for the different age groups in China was derived from the World Health Organization website (World Health Organization 2012) and was used to calculate all-cause mortality for the model.

#### Cost inputs

The various types of costs included were health state costs, drug costs, costs related to the management of renal impairment, and diagnostic costs (Table 6). The health state costs were primarily obtained from a study by Zhang et al. (2015). The drug costs were obtained from the IMS PADDS 2015 database. At the time of this evaluation, ETV, LAM, and ADV were available as generics, and hence, their generic costs were included, whereas for LDT and TDF, the branded costs were used. All values were presented in US dollars (USD or $), and wherever not available in USD, Ren Min Bi (RMB) was converted to USD at a rate of 6.22 RMB per USD.

## Results

### Deterministic results

**Fig. 2:**
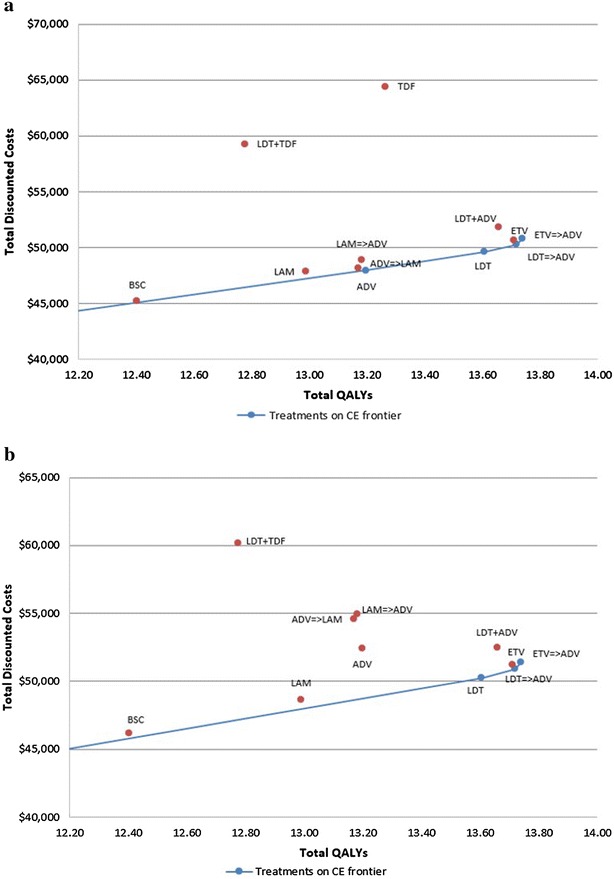
Cost-effectiveness frontier **a** without renal impact and **b** including renal impact. *ADV* adefovir dipivoxil, *BSC* best supportive care, *CE* cost-effectiveness, *ETV* entecavir, *LAM* lamivudine, *LDT* telbivudine, *QALY* quality-adjusted life-year, *TDF* tenofovir

Total discounted costs of the various treatment strategies (Table 7) were interpreted using the cost-effectiveness frontier (Fig. 2a, b). Cost-effectiveness frontier is a chart with the total discounted QALYs plotted along the x-axis and the total discounted costs along the y-axis. Every treatment is depicted on the chart with its total discounted costs and total discounted QALYs. In general, a treatment strategy appearing at the bottom right quadrant signifies that it generated large QALYs at a lower cost, whereas that located at the top left quadrant signifies that it generated few QALYs at a considerably high cost. The blue line connects the cost-effective treatments. Treatment options that lie above this line are not considered cost effective. At present, willingness to pay (WTP) in China is estimated to be approximately $23,000 (3 times the gross domestic product [GDP] of China) (World Bank National Accounts Data 2015; Zhang et al. 2015).

**Table 7:** Total discounted costs and QALYs for the treatment strategies

Treatment strategy	Cost-effectiveness analysis results without considering renal impact			Cost-effectiveness analysis results considering renal impact		
	Cost ($)	QALYs	ICER with respect to next best option	Cost ($)	QALYs	ICER with respect to next best option
*Without sequencing*						
BSC	45,234	12.40	–	46,171	12.40	–
LAM	47,838	12.99	ED	48,679	12.99	ED
LDT	49,620	13.60	4066	50,257	13.60	3398
ADV	47,963	13.20	3435	52,423	13.20	D
ETV	50,640	13.71	D	51,248	13.71	D
TDF	64,413	13.27	D	65,291	13.27	D
LDT + ADV	51,829	13.66	D	52,446	13.66	D
LDT + TDF	59,267	12.78	D	60,114	12.78	D
*With sequencing*						
LAM → ADV	48,878	13.18	D	54,976	13.18	D
ADV → LAM	48,231	13.17	D	54,560	13.17	D
LDT → ADV	50,275	13.72	5774	50,868	13.72	5385
ETV → ADV	50,819	13.74	27,205	51,422	13.74	27,741

*ADV* adefovir dipivoxil, *BSC* best supportive care, *D* dominated, *ED* extended dominance, *ETV* entecavir, *ICER* incremental cost-effectiveness ratio, *LAM* lamivudine, *LDT* telbivudine, *QALY* quality-adjusted life-year, *TDF* tenofovir

When renal impact was not considered, without treatment sequencing, ADV and LDT were found to be more cost effective compared with other NUCs (Fig. 2a). The overall costs, QALYs and incremental cost-effectiveness ratios (ICERs) are presented in Table 7. Amongst the strategies with treatment sequencing, LDT → ADV was cost effective. Interestingly, ETV → ADV sequence may appear cost effective as it generates higher QALYs; however, this strategy has a high ICER ($27,205) which is above the acceptable WTP in comparison to the LDT → ADV strategy.

When renal impact was considered, LDT and LDT → ADV strategies (with or without sequencing) appeared to be better in terms of cost effectiveness. For example, the ICER of LDT to BSC was lowered from $4066 to $3398 (Table 7).

### Probabilistic sensitivity analysis (PSA)

**Fig. 3:**
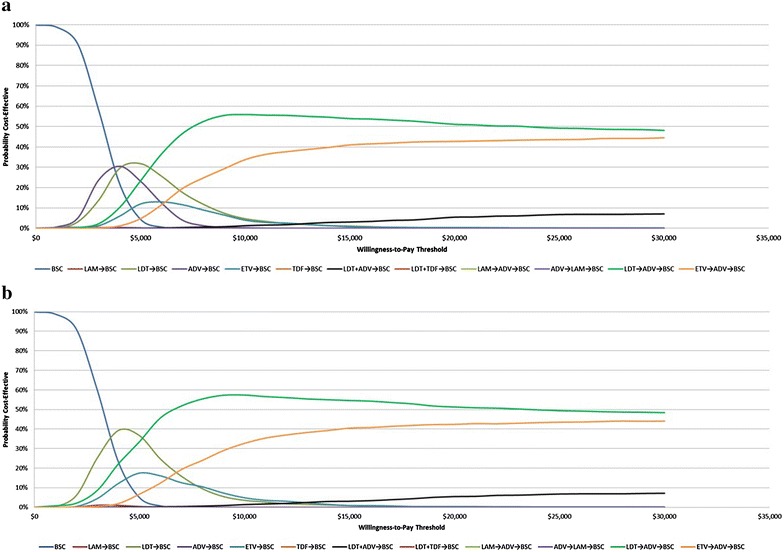
Cost-effectiveness acceptability curve **a** without renal impact and **b** with renal impact. *ADV* adefovir dipivoxil, *BSC* best supportive care, *ETV* entecavir, *LAM* lamivudine, *LDT* telbivudine, *TDF* tenofovir

The uncertainty of the cost-effectiveness results for a range of WTP thresholds were interpreted using cost-effectiveness acceptability (CEAC) curves. For both scenarios, LDT → ADV sequence was cost effective with 51 % probability even at willingness to pay of $20,000. The next best scenario was the combination treatment of ETV and ADV, which was cost effective with approximately 43 % probability (Fig. 3a, b).

## Discussion

CHB is one of the most common causes of chronic liver disease, cirrhosis and HCC worldwide (Lavanchy 2004; McMahon 2005). In CHB patients, glomerulonephritis is an important extrahepatic manifestation of the viral infection (Chan 2010). Particularly, with aging, several patients present with comorbidities and various degrees of functional renal impairment (Deterding et al. 2011; Ha et al. 2009; Lai et al. 1991). Since the clinical management of CHB is mainly based on NUC therapy, there is a need for preservation of renal function in these patients. Such an outcome could be achieved by inclusion of NUCs with minimal nephrotoxicity in the treatment strategy. Especially in an evolving healthcare setting such as China, where real-world use of NUCs is primarily driven by costs along with clinical efficacy evidence (Fung et al. 2011), treatment strategy may be guided by robust pharmacoeconomic evidence.

Previous studies have reported ETV as the most cost-effective NUC and advocated its use as a first-line antiviral therapy/preferred treatment option in patients with CHB (Wu et al. 2010; Wu et al. 2012) in Chinese healthcare settings. However, treatment and disease-related transition rates in these studies were primarily obtained from the literature using cohorts from different countries, which may affect the generalizability of the results and may not accurately reflect the situation in China. Moreover, these studies did not consider the long-term clinical outcomes associated with use of NUCs e.g. renal impact or development of drug resistance.

A recent study by Zhang et al. used a more systematic approach to identify the registered clinical trials that were based on Chinese populations and conducted meta-analyses to derive the parameter inputs (Zhang et al. 2015). The present model included inputs used by Zhang et al., to ensure that parameters reflect a real-world Chinese patient population. Long-term renal complications and drug resistance were modeled in this patient cohort, and combination and sequence therapies of NUCs were considered in the analysis. In addition to including similar model inputs as those in the study by Zhang et al., the present study also included additional model parameters such as resistance profiles of NUCs and annual changes in eGFR with various treatments. Thus, the findings of the present analysis more closely reflect the clinical conditions of Chinese patients in real-world settings.

In the study by Zhang et al., treatment with ETV generated the highest number of QALYs, resulting in 10.8 QALYs compared with the next best result of 9.8 QALYs with LDT (Zhang et al. 2015). The present analysis showed QALY gain of 0.02 for ETV versus LDT (Table 7). In our model, there is a reduced difference in the rate of virologic resistance between LDT and ETV. Therefore, patients stayed longer on LDT compared with Zhang’s analysis, and accordingly more patients moved to the inactive carrier state with LDT treatment. The benefit of ETV in terms of lesser resistance was apparently offset by LDT’s impact on moving patients to the inactive carrier state. Thus, both regimens appeared similar in terms of QALYs in the current analysis. Another factor that contributed to QALY gain in Zhang et al. was virologic response. As ETV had a higher virologic response than LDT, patients spent more time in the response state, thereby contributing to QALYs. This factor was not considered in our model.

In a real-world Chinese healthcare setting, treatment with NUCs leads to the development of resistance, resulting in a switch to the next best therapy (Chinese Society of Hepatology and Chinese Society of Infectious Diseases and Chinese Medical Association 2011). The results of this model indicated that LDT → ADV was the most cost-effective treatment strategy. Other real-world studies in Chinese populations have also reported findings that support the results of this pharmacoeconomic analysis. In a retrospective study conducted in CHB patients from China, the renoprotective effect of LDT was found to be superior to that of ADV when both were used as monotherapies for 1 year (Li et al. 2012). Similar findings were reported in other studies in the Chinese population (Gane et al. 2014; Wang et al. 2013). Furthermore, a recent prospective cohort study provided evidence that in the Chinese population, prolonged LDT therapy resulted in an improved eGFR, whereas ADV therapy was associated with a decreased eGFR and both LAM and ETV therapies did not significantly influence eGFR (Qi et al. 2015). As renal protection is an important treatment-related concern in CHB patients (Deray et al. 2015), LDT offers clinically relevant efficacy and has a safety profile that may make it a possible therapeutic option for high-risk patients. In addition, clinical practice guidelines recommend the use of LDT in patients at an increased risk of renal impairment (Deray et al. 2015). The findings of the present analysis are in congruence with the real-world evidence (Gane et al. 2014; Wang et al. 2013), confirming the favorable clinical profile of LDT compared with other NUCs because of its lower renal toxicity. The findings of this study would enable Chinese payers to make evidence-based justifiable decisions.

### Limitations

The present model has several limitations. The methodology adopted in this analysis was that of a transition state model that focused on HBeAg seroconversion. This approach is entirely based on the observations from HBeAg-positive patients and may not be relevant for HBeAg-negative patients. For a few safety inputs, particularly eGFR, the evidence was sparse, and the numeric values used in the model were not derived from a meta-analytic synthesis. Furthermore, disutility due to renal function is expected to be different for patients in a CHB state versus patients in a decompensated cirrhosis state. Because of the lack of granular data, the current study could not model the differential utilities for patients in a CHB state versus those in decompensated cirrhosis state. Thus, the current analysis represents a conservative assessment or an underestimate of the cost effectiveness of LDT given its association with improved renal function.

## Conclusion

In this pharmacoeconomic evaluation, LDT treatment proved to be cost effective for CHB in Chinese healthcare settings. Considering the impact of NUCs on renal function, overall costs for all evaluated treatment options were increased. However, the increase for LDT was comparatively small.
